# iTRAQ‐based quantitative proteomics analysis of immune thrombocytopenia patients before and after Qishunbaolier treatment

**DOI:** 10.1002/rcm.8993

**Published:** 2020-12-03

**Authors:** Burenbatu, Yanbo Wang, Shuanglian Wang, Narisu, Wuritunashun, Cuiqin Gong, Hashengaowa, Eerdunduleng, Sarula, Guihua, Haihua Bai

**Affiliations:** ^1^ Affiliated Hospital of Inner Mongolia University for Nationalities Tongliao Inner Mongolia 028000 China; ^2^ College of Life Sciences and Food Sciences Inner Mongolia University for Nationalities Tongliao Inner Mongolia 028000 China; ^3^ Inner Mongolia Engineering and Technical Research Center for Personalized Medicine Inner Mongolia University for Nationalities Tongliao Inner Mongolia 028000 China

## Abstract

**Rationale:**

Treatment of immune thrombocytopenia (ITP) usually involves long‐term use of immunosuppressive corticosteroids and splenectomy. However, these treatments often have side effects in patients. The Mongolian medicine Qishunbaolier (QSBLE) has a high curative effect, reduces the chances of relapse, and has no obvious side effects. This study was designed to identify potential therapeutic targets of QSBLE for treating ITP.

**Methods:**

To reveal differences in protein expression between ITP patients (ITPs) before and after QSBLE treatment, comparative proteomics studies were performed using isobaric tags for relative and absolute quantification (iTRAQ). The analysis used nanospray liquid chromatography/tandem mass spectrometry (nano‐LC/MS/MS) in positive ion electrospray ionization mode. Key proteins relevant to ITP were revealed by the Kyoto Encyclopedia of Genes and Genomes (KEGG) and other bioinformatics tools. Real‐time polymerase chain reaction (RT‐PCR) analysis was carried out for confirmation of differentially expressed proteins.

**Results:**

A total of 982 differentially expressed proteins were identified in ITPs compared with the controls. Compared with the pre‐QSBLE treatment group, 61 differentially expressed proteins were identified in the post‐QSBLE treatment group, with 48 proteins being significantly upregulated and 13 downregulated. Twenty‐nine pathways were significantly enriched. Q6N030 and other proteins were the key players in the protein‐pathway network. Twenty proteins that may play important roles in the treatment of ITP were further filtered. RT‐PCR and Western blot analyses further confirmed that MIF, PGK1 and IGHM were upregulated in ITPs after QSBLE treatment, in accordance with the proteomics data.

**Conclusions:**

It is believed that the identified proteins and the results of bioinformatics analysis will provide a potential therapeutic target site for QSBLE for ITP therapy and biomarkers.

## 1 INTRODUCTION

Immune thrombocytopenia (ITP) is an autoimmune disease with an increased tendency of hemorrhage, characterized by increased cell and antibody‐mediated platelet destruction, purpura, ecchymosis, and mucosal bleeding.^1^ ITP has a disease group in people of different ages, genders, and races. The age of onset is polarized, with higher incidence in children under 14 years of age and older people over 60 years of age.^2^, ^3^ ITP can be asymptomatic or can present with petechiae, ecchymoses, mucocutaneous bleeding, nasal bleeding, or serious bleeding into the intestine or brain.^4^ Intracranial hemorrhage occurs in <1% of ITP patients (ITPs) but it is a life‐threatening complication of the disease.^5^ Although an enhanced tendency of bleeding is a typical clinical feature of ITP, this disease is paradoxically associated with thromboembolic events.^6^ Recently, new drugs for thrombopoietin receptor antagonists have been used to treat chronic ITP. However, these methods often have adverse reactions during treatment, including infection, bone marrow suppression or fibrosis, and recurrence.^7^, ^8^ In addition, corticosteroids usually have a therapeutic effect in only 70% of patients, while the alternative splenectomy surgical treatment increases the risk of developing internal bleeding.^8^, ^9^ Based on these side effects, corticosteroids and splenectomy should only be used carefully. Searching for a highly effective, low‐rebound, and safer drug is an active direction for the treatment of this disease.

Mongolians have long used natural products, including herbs, animals, and minerals, to fight diseases in their nomadic and hunting life. Since the 1960s, Mongolian medicines have been used to treat a large number of diseases, including ITP, aplastic anemia (AA), allergic purpura (AP), and other bleeding disorders such as myelodysplastic syndrome (MDS).^10^ Qishunbaolier (QSBLE) is a Mongolian preparation containing gardenia, bezoar, cornu bubali, cattle gall powder, saffron, lithospermum, and rubia.^10^ Toxicity studies have shown that QSBLE has no toxic side effects on peripheral blood mononuclear cells or on the liver, heart, and kidney in mice; no obvious symptoms were found after high‐dose administration.^10^ Previously, we profiled miRNA expression in ITPs before and after QSBLE treatment and identified 44 miRNAs that were differentially expressed.^11^ Of these 44 miRNAs, 25 were downregulated in ITPs.^11^ These 25 miRNAs may be closely related to the pathogenesis of ITP. However, the impact of changes in protein expression before and after QSBLE treatment needs further study. Proteomics is an effective tool for the characterization of protein expression profiles, and has been widely used to study protein‐related diseases.^12^, ^13^ Therefore, further identification and exploitation of potential molecular biomarkers will contribute to the prevention and treatment of ITP.

Our study aimed to use isobaric tags for relative and absolute quantification (iTRAQ) methods to determine the presence of differentially expressed proteins and biological pathways between ITPs before and after QSBLE treatment. Our results provide a comprehensive list of proteins associated with the progression of ITP; these may prove to be useful as potential markers or therapeutic targets.

## 2 MATERIALS AND METHODS

### 2.1 Patient materials

This study was approved by the Institutional Review Board of the Affiliated Hospital of Inner Mongolia University for Nationalities, and is in line with the Helsinki Declaration. Written informed consent was obtained from each participant. Chronic ITPs were diagnosed after examination by the Inner Mongolia University for Nationalities Hospital. The diagnostic criteria for chronic ITP meet the guidelines for diagnosis and treatment.^14^ These include no increase or a slight increase in the spleen, a decrease in platelet count, an increase in bone marrow megakaryocytes or a normal increase in number, but no maturation of thrombocytopenia. In addition, at least one of the following five criteria should be met during the diagnosis of ITP: reactive with prednisone, effective splenectomy, increase in platelet‐associated IgG, shortened platelet life, or increased platelet‐associated complement components C3.

To rule out other complications, patients enrolled in the study needed to undergo standard health checks for health disorders, including hypertension, cancer, infection, diabetes, and other systemic diseases, before being diagnosed with ITP. During the treatment, ITPs were provided with 3–5 g QSBLE (weight‐based) per day. The efficacy of QSBLE was assessed by tracking platelet counts and bleeding scores, and tracking ITP treatment every 3 weeks for up to 12 weeks.

### 2.2 Sample collection and processing

In order to quantify protein sample preparation for mass spectrometry analysis, serum was depleted of most abundant proteins using a Human 14 multiple affinity removal system column (Agilent Technologies, Santa Clara, CA, USA) following the manufacturer's protocol. A 10 kDa ultrafiltration tube (Sartorius, Göttingen, Germany) was used for desalination and concentration of low‐abundance components. One volume of SDT (2% sodium dodecyl sulfate (SDS), 100 mM dithiothreitol (DTT), 100 mM Tris‐HCl, pH 7.6) buffer was added, and the solution was then boiled for 15 min and centrifuged at 14000 *g* for 20 min. The supernatant was quantified using a BCA Protein Assay Kit (Bio‐Rad, Hercules, CA, USA). The samples were stored at −80°C.

### 2.3 iTRAQ labeling and peptide fractionation

Equal amounts of protein in the pre‐ and post‐QSBLE treatment ITP groups, and the control group, were denatured with 2% SDS, followed by reduction with 5 mM DTT, and alkylation with 20 mM iodoacetamide. In addition, modified sequencing grade trypsin (Promega, Madison, WI, USA) was digested overnight at 37°C in a 1:20 enzyme‐to‐substrate ratio. The peptide chain was labeled with iTRAQ reagent using a 4/8‐plex Multiplex reagent kit, (AB SCIEX, Foster City, CA, USA) according to the manufacturer's instructions. Three biological replicates were prepared for all samples. For the biological replication, three 8‐plex iTRAQ sets were used. The samples were labeled as 113 (normal), 114 (effective treatment in ITP patients before QSBLE treatment), 115 (invalid treatment in ITP patients before QSBLE treatment), 116 (effective treatment in ITP patients after QSBLE treatment), and 117 (invalid treatment in ITP patients after QSBLE treatment). All labeled samples were then combined and dried under vacuum.

A 100 μg peptide mixture of each sample was labeled using an iTRAQ reagent according to the manufacturer's instructions (Thermo Fisher Scientific, Waltham, MA, USA). The iTRAQ‐labeled peptides were fractionated^15^ by reversed‐phase (RP) chromatography using an Agilent 1260 infinity II HPLC system. The peptide mixture was diluted with buffer A (10 mM HCOONH_4_, 5% acetonitrile (can), pH 10.0), and loaded onto a XBridge Peptide BEH C18 column (130 Å, 5 μm, 4.6 mm × 100 mm; Waters, Milford, MA, USA). The peptides were eluted at a flow rate of 1 mL/min with a gradient of 0% buffer B (10 mM HCOONH_4_, 85% ACN, pH 10.0) for 25 min, 0%–7% B during 25–30 min, 7%–40% B during 30–65 min, 40%–100% B during 65–70 min, and 100% B during 70–85 min. The elution was monitored at 214 nm, and fractions were collected every 1 min during 30–65 min. The collected fractions were dried down via vacuum centrifugation and dissolved with 0.1% formic acid (FA).

### 2.4 Nano‐LC/MS/MS analysis

Each sample was separated using a nanoliter flow rate Easy nLC system (Thermo Fisher Scientific). Buffer A was a 0.1% FA aqueous solution, and buffer B was a 0.1% FA/80% ACN aqueous solution. The column was equilibrated with 100% buffer A and the sample was loaded onto an analytical column (Acclaim PepMap RSLC; 50 μm × 15 cm, nano viper, P/N 164943; Thermo Fisher Scientific) using an autosampler at a flow rate of 300 nL/min. A 1‐h gradient was programmed as follows: 0 min–5 min, linear gradient of buffer B from 0% to 6%; 5 min–45 min, linear gradient of B from 6% to 28%; 45 min–50 min, linear gradient of B from 28% to 38%; 50 min–55 min, linear gradient of buffer B from 38%–100%; 55 min − 60 min; and then maintained at 100% B. The separated compounds were analyzed by positive ion electrospray ionization mass spectrometry using a Q‐Exactive hybrid Quadrupole‐Orbitrap mass spectrometer (Thermo Fisher Scientific). The scan range was *m/z* 350–1800, the mass resolution was 70,000, the automatic gain control (AGC) target was set at 3e6, and the maximum ion injection time was 50 ms. The 10 most abundant ions in each full scan were then selected for higher‐energy collisional dissociation (HCD) to generate their respective product ion spectra, using a precursor ion isolation window of 2 *m/z* units. For these experiments the resolution was 17,500, the number of microscans was 1, the maximum injection time was 45 ms, and the normalized collision energy was 30 eV.

### 2.5 Nucleic acid isolation and real‐time (RT)‐PCR

RNA isoplus (Cat. No. 9112; Takara, Shiga, Japan) was used to extract RNA from white blood cells before and after treatment in ITPs and normal controls. A PrimeScript™ RT reagent kit (Cat. No. RR047A; Takara) was used for reverse transcription, following the manufacturer's instructions. RT‐PCR (Cat. No. RR820A; Takara) was performed to quantify relative gene expression. GAPDH was used as an endogenous control. The relative concentration of candidate gene expression was calculated as described in the SYBR Green user manual (Thermo Fisher Scientific), using the formula 2^‐ΔΔCT^. The primers used for the RT‐PCR are listed in Table S1 (supporting information).

### 2.6 Western blot analysis

Western blot analysis was performed as described previously.^16^ A nanodrop was used to test the concentrations of the protein. The proteins underwent electrophoresis in a 12% SDS‐PAGE gel and were then transferred to a nitrocellulose membrane. After blocking with 4% BSA, the primary antibody was incubated overnight at 4°C. The primary antibody was incubated for 1 h at room temperature. The immunoglobulin heavy constant mu (IGHM) monoclonal antibody (Proteintech, Rosemount, IL, USA) was used at 1:5000, the macrophage migration inhibitory factor (MIF) monoclonal antibody (R&D Systems, Minneapolis, MN, USA) was used at 1:10000, and the phosphoglycerate kinase 1 (PGK1) polyclonal antibody (Invitrogen, Carlsbad, CA, USA) was used at 1:10000. The secondary antibody was incubated with IgG‐horseradish peroxidase (HRP) labeled for 1 h.

### 2.7 Data analysis

The raw mass spectrometry data were acquired in a RAW file, and subjected to library identification and quantitative analysis using Mascot 2.5 and Proteome Discoverer software (version 2.1; Thermo Fisher Scientific Inc., Bremen, Germany). Identification and analysis were performed using human Uniprot protein databases including known contaminants (Database version: HomoSapiens, 137,207 entries, 05‐Dec‐2016). Search parameters included trypsin as an enzyme, allowing up to 2 misses of cutting. The oxidation of methionine was set to dynamic modification, while the carbamoylation of cysteine, the N‐terminus of the peptide, and the iTRAQ modification of lysine were set to static modification. The precursor and fragment mass tolerances were set to 10 ppm and 0.05 Da, respectively. A 1% false discovery rate of peptide levels was used as a filter for peptide identification. The reporter ion quantification node was used for determining the relative expression pattern of the protein based on the relative intensity of the reporter ions of the corresponding peptide. Proteins that increase or decrease the ratio by 1.2‐fold or more between different groups are considered to be upregulated or downregulated, respectively. A protein‐pathway network analysis was carried out using Cytoscape, as described previously.^17^ The protein‐pathway network was prepared based on the differential protein before and after treatment of the ITPs. All the pathways with P <0.05 in the KEGG and the proteins involved in the pathway were visualized with Cytoscape software (www.cytoscape.org).

### 2.8 Statistical analysis

The ITP data pre‐ and post‐QSBLE treatments were compared using the Mann–Whitney T‐test in GraphPad (San Diego, CA, USA) Prism 5.0 software. When the P‐value was <0.05, the difference was considered to be significant.

## 3 RESULTS

### 3.1 Characteristics of the study participants

Patients with chronic ITP were diagnosed by the Affiliated Hospital of Inner Mongolia University for Nationalities. The study was approved by the Ethics Committee of the Affiliated Hospital of Inner Mongolia University for Nationalities. The number of platelets in normal subjects was 100 × 10^9^/L–300 × 10^9^/L. The ITPs for this study were individuals who had chronic ITP due to bone penetration, and had a platelet count between 10 × 10^9^/L and 100 × 10^9^/L. There were no other secondary factors from thrombocytopenia, no obvious bleeding tendency, and patients had no liver or kidney dysfunction. Of the 18 ITPs, 10 were women and 8 were men. The ITPs were between 19 and 55 years old. Clinical information of all the patients is listed in Table S2 (supporting information). The patients were divided into an effective treatment group and an invalid treatment group, with the average age of 39.56 and 35.56, respectively. Among the 9 participants receiving the effective treatment, 5 were men and 4 were women. In the invalid treatment group, 3 were men and 6 were women (Table S3, supporting information). In this study, the effective treatment standards for ITPs met the following criteria: decreased platelets returned to normal or increased by 30 × 10^9^/L after treatment. The results showed that patients taking QSBLE showed an increase in the platelet count from 46.9 × 10^9^/L to 115.2 × 10^9^/L, which effectively treated ITP (Table 1). Further observations upon closely following these QSBLE patients for up to 12 weeks did not reveal any side effects usually associated with glucocorticoid therapy, including loss of appetite, vomiting, increased heart rate, nausea, increased sweating, difficulty in sleeping or any other symptoms. We did not observe any significant recurrence after QSBLE treatment (data not shown).

**TABLE 1:** Platelet count before and after QSBLE treatment

Group	Platelet count (×10^9^/L)	T‐test	P‐value
ITPs (n = 9)	46.89 ± 17.19	−5.911	0.035*
Treatments (n = 9)	115.22 ± 44.68		

*The significant difference was detected by a T‐test.

### 3.2 Differentially expressed proteins in ITPs before and after QSBLE treatment

**FIGURE 1:**
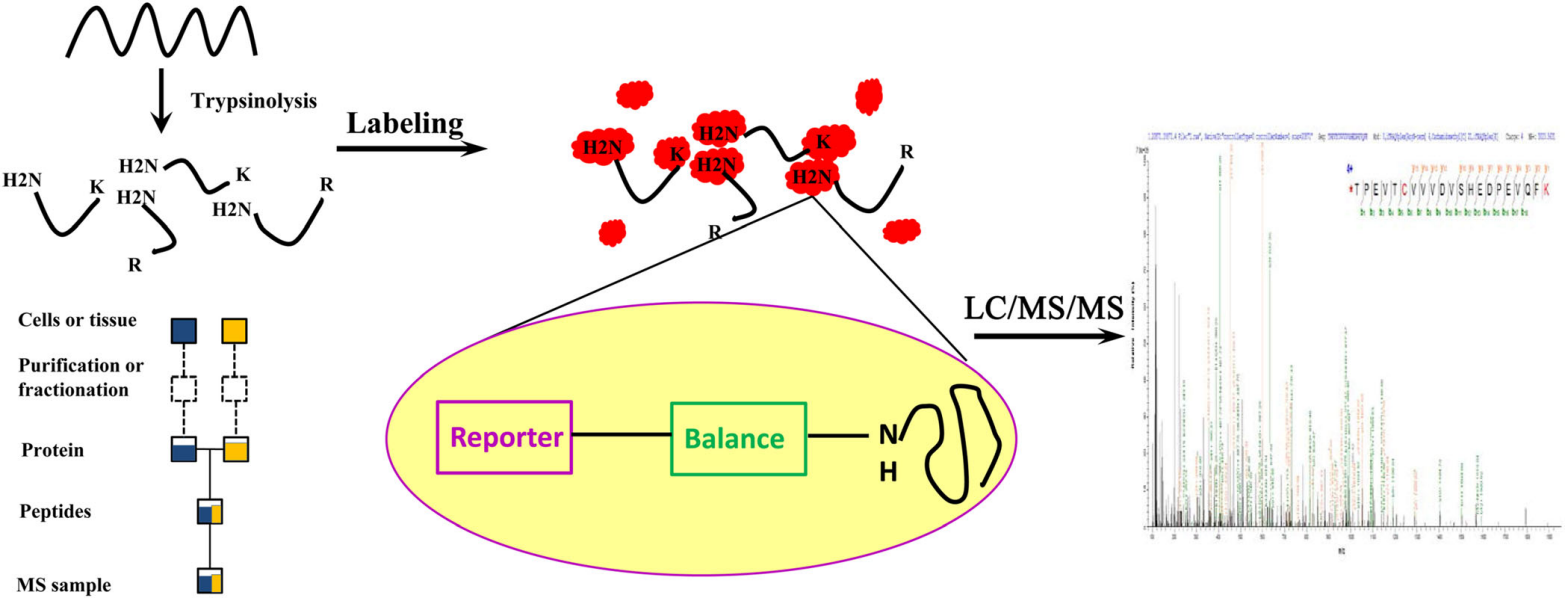
Schematic representation of the workflow of ITP proteins. The iTRAQ‐based strategy was employed for the comparison of the proteome from ITP patients (ITPs) before and after QSBLE treatment

In order to study and compare the differentially expressed proteins in the blood of ITPs and controls, protein samples were labeled with iTRAQ reagents and analyzed by nano‐LC/MS/MS (Figure 1). In our study, a total of 5717 unique peptide groups belonging to 982 proteins were identified (Table S4, supporting information). Proteins that showed significant changes (±1.2‐fold or more) in the ITPs before and after QSBLE treatment were considered as differentially expressed. P values <0.05 were considered statistically significant. Sixty‐one differentially regulated ITP proteins were identified, and are listed in Table S5 (supporting information). Forty‐eight proteins were found to be overexpressed in ITPs before QSBLE treatment, whereas 13 proteins were downregulated after QSBLE treatment (Table 2). Similarly, there were 261 differentially expressed proteins between pre‐treatments and healthy subjects (Controls) (Table 2). Of these proteins, 48 were upregulated and 213 were downregulated in ITPs before treatment with QSBLE compared with controls (Table 2). We also compared the differentially expressed proteins pre‐ and post‐treatment in the invalid treatment group. Interestingly, in that group, the number of differentially expressed proteins was lower than in the effective treatment group. Of these proteins, 23 were upregulated and 13 were downregulated in the invalid treatment ITPs pre‐ and post‐treatment with QSBLE (Table 2). The peptide identification information, including the Peptide‐Spectrum Match (PSM), the [M + H]^+^ precursor ions, and the precursor ion charge, is listed in Table S6 (supporting information).

**TABLE 2:** Number of significantly differentially expressed proteins identified by iTRAQ analysis of ITPs before and after QSBLE treatment

Comparisons	Up‐	Down‐	All‐
B/A	48	213	261
D/B	48	13	61
E/C	23	13	36

(A) Controls (healthy individuals), (B) effective treatment ITPs before QSBLE treatment, (C) invalid treatment ITPs before QSBLE treatment, (D) effective treatment ITPs after QSBLE treatment, and (E) invalid treatment ITPs after QSBLE treatment.

### 3.3 Gene ontology analysis of identified proteins

**FIGURE 2:**
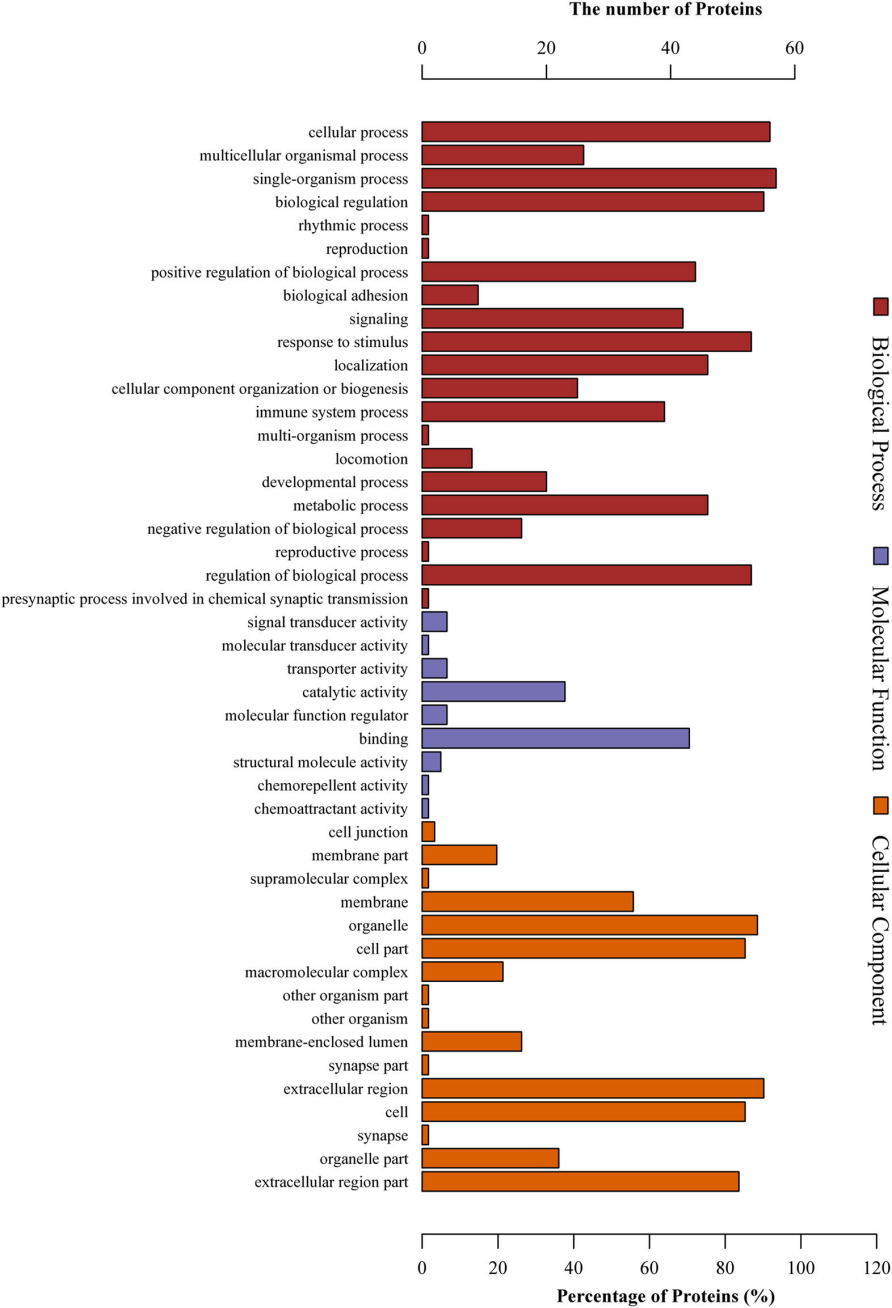
Gene ontology (GO) analysis of the proteins in ITPs. GO annotation was achieved using Blast2GO. Protein sequences were grouped into three categories based on their properties and functions

Proteins that were identified only in ITP patients before or after QSBLE treatment were found to differ in their characteristics. Gene ontology (GO) enrichment indicated that these differentially expressed proteins were mainly involved in biological processes such as biological regulation, biological adhesion, immune system process, signaling, locomotion, and developmental process (Figure 2). In terms of molecular function annotation, these differentially expressed proteins were mainly involved in signal transducer activity, molecular transducer activity, transporter activity, molecular function regulator, binding, structural molecular activity and chemorepellent activity (Figure 2). In terms of molecular function cellular and component annotation, these differentially expressed proteins were mainly involved in the cell junction, membrane, organelle, cell part, macromolecular complex, membrane‐enclosed lumen, extracellular region, and cell and organelle part (Figure 2). The results indicated that these proteins were related to immune response which may make them potential biomarkers for ITP diagnosis.

### 3.4 KEGG pathway analysis

**FIGURE 3:**
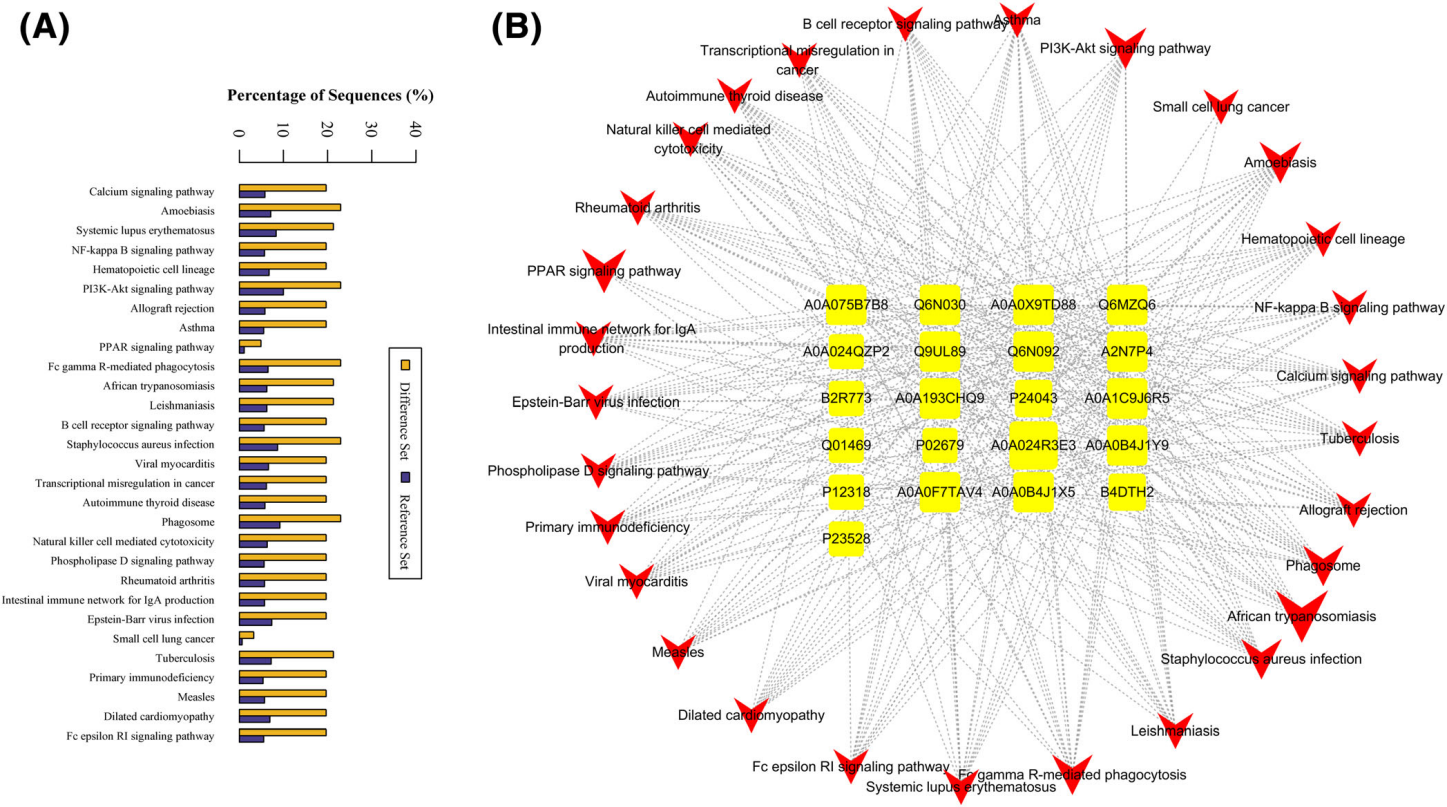
KEGG enrichment annotation of the differentially expressed proteins and protein‐pathway network of QSBLE against ITP: A, each column represents a pathway and B, the topological analysis of 29 pathways and 21 proteins was carried out with betweenness centrality. The yellow squares represent target proteins and the red V‐shapes represent pathways

The KEGG pathway is a utility database resource for understanding advanced functions and biological systems (such as cells, organisms, and ecosystems), genome sequencing from molecular‐level information, especially large molecular data sets, and other high‐throughput experimental techniques. Pathway enrichment analysis was also performed by searching against the KEGG database. According to the results of the KEGG enrichment analyses, 29 pathways were included in the differentially expressed proteins (Figure 3A). The results showed that most of the abundant KEGG terms were mainly involved in biological processes such as the calcium signaling pathway, NF‐κB signaling pathway, phospholipase D signaling pathway, PI3K‐Akt signaling pathway, hematopoietic cell lineage, natural killer cell mediated cytotoxicity, B cell receptor signaling pathway, Fc epsilon RI signaling pathway, Fc gamma R‐mediated phagocytosis, intestinal immune network for IgA production, leishmaniasis, African trypanosomiasis, amebiasis, *Staphylococcus aureus* infection, tuberculosis, measles, transcriptional misregulation in cancer, asthma, autoimmune thyroid disease, systemic lupus erythematosus, rheumatoid arthritis, allograft rejection, primary immunodeficiency and viral myocarditis. (Figure 3A). In order to further analyze the potential target proteins in ITP therapy in the KEGG signaling pathway, we constructed a protein‐pathway network (Figure 3B). The results revealed that these differentially expressed proteins were highly associated with immune diseases, and immune system development and function, such as the Fc epsilon RI signaling pathway and immune network for IgA production are potential therapeutic target proteins for ITP.

### 3.5 MS analysis in ITP pathogenesis and gene expression verification

**FIGURE 4:**
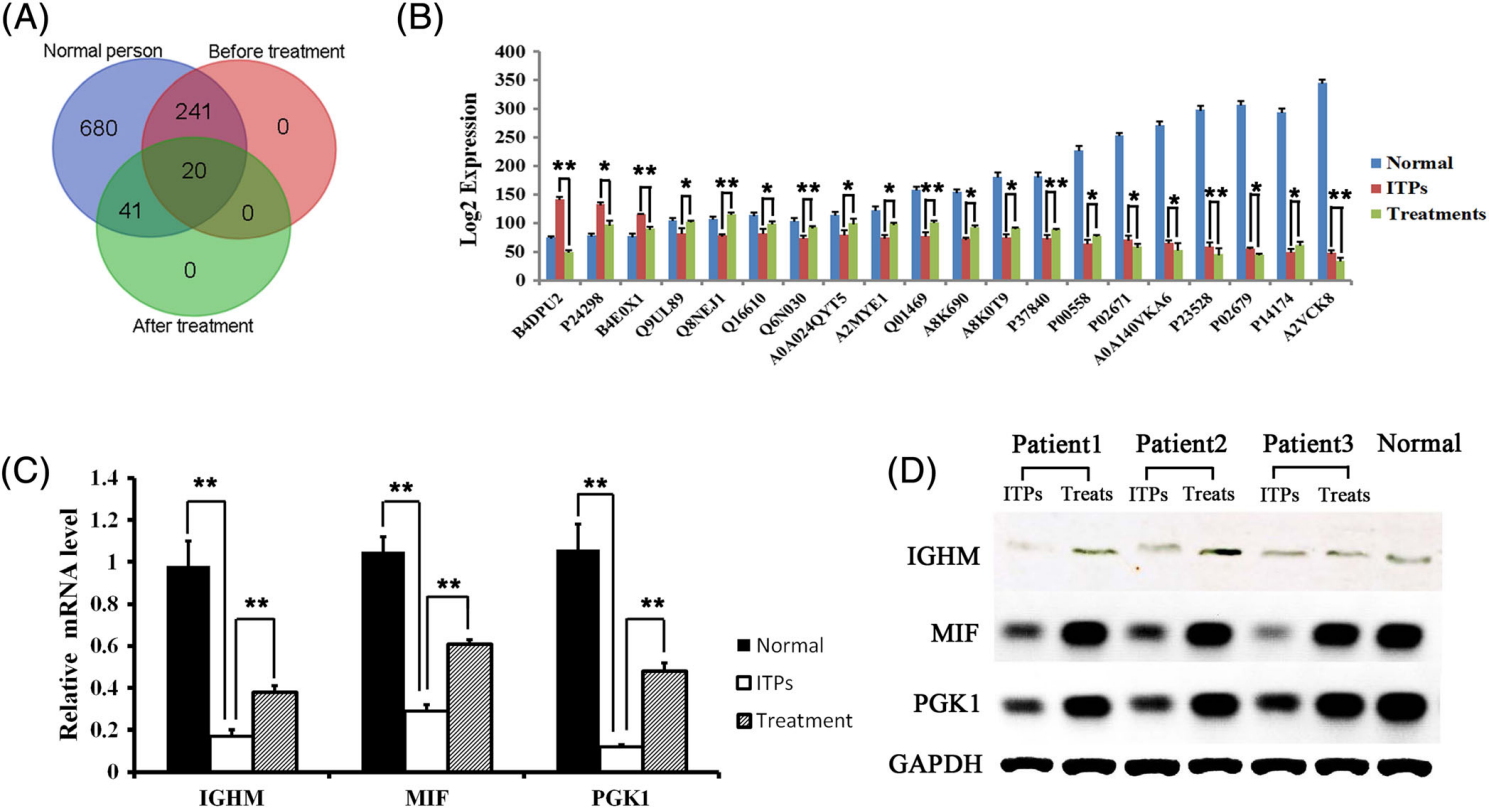
Venn maps of the 20 identified proteins and gene expression confirmation of IGHM, MIF, and PGK1. Twenty significantly differentially expressed proteins filtered by expression in normal people, ITP patients (ITPs), and ITPs after treatment. A, Multiple sets of iTRAQ‐tagged protein Venn maps. B, Log2 expression of the 20 identified protein. Each sample contains the blood of 3–5 individuals. The test is repeated three times for each sample. C, Real‐time PCR results of IGHM, MIF, and PGK1. D, Western blot results of IGHM, MIF, and PGK1. Statistical data are presented as the mean ± SEM. *P <0.05; **P <0.01

It is unclear whether protein dysregulation is associated with the pathogenesis of ITP. We conclude that ITP‐associated proteins are abnormally expressed in ITPs, but can be restored to control levels after QSBLE treatment. To further screen for functional proteins, we reanalyzed differentially expressed proteins and focused on proteins that are abnormally expressed in ITP which are also expressed in control and treatment. Finally, we identified 20 proteins that met these criteria (Figure 4A, and Table S5, supporting information). Of these 20 proteins, P14174, P00558 and Q6N030, which are encoded by MIF, PGK1 and IGHM, respectively, were closely related to immunity.^18^, ^19^, ^20^ The results of the RT‐PCR and Western blot analyses showed that the expression of these three genes was upregulated in ITPs after QSBLE treatment, which is consistent with the proteomics results (Figures 4C and 4D). When MIF is inhibited, it usually leads to a functional reversal from immunosuppressive MDSCs to immunostimulatory dendritic cells (DCs), some of which may be due to a decrease in MDSC prostaglandin E2 (PGE2).^18^ In a recent study, PGK1 was used as an immune target in Kawasaki disease.^19^ Genetic studies have shown that patients affected by ARA due to μ heavy chain defects are complex heterozygotes of the IGHM gene deletion.^20^ The three genes were all upregulated in the ITPs after treatment compared with those before treatment (Figure 4B, and Table S5, supporting information). This indicates that these three proteins might be used as biomarkers for ITP diagnosis.

## 4 DISCUSSION

ITP is an autoimmune disorder defined by low platelet count under the level of 100 × 10^9^/L, secondary to increased peripheral platelet destruction by autoantibodies. Some studies have reported increased risks of thromboembolic events in adults with ITP but thromboembolic events after intravenous immunoglobulin (IVIG) treatment in pediatric ITP are very rare, and only five cases have been reported to date.^21^, ^22^, ^23^, ^24^ Traditionally, macrophage and autoantibody‐mediated phagocytosis in the spleen play a major role in platelet destruction in the pathogenesis of ITP.^25^, ^26^ At present, the source of these antibodies is not clear, but recent studies have shown that when infected with hepatitis virus or HIV virus, it is easy to induce anti‐autoreactivity, which in turn stimulates the production of anti‐platelet antibodies.^27^ Studies have also shown that T cells play an important role in the immune pathogenesis of ITP. Because T cells have a self‐tolerance that tends to be self‐reactive, dysregulation of T cells is crucial in the development of ITP.^28^, ^29^, ^30^, ^31^

There are many factors that can cause the pathogenesis of ITP, including abnormalities of various immune cells. Among them, the B‐cell‐activating factor (BAFF) in the tumor necrosis factor (TNF) family is an important part of normal B‐cell homeostasis.^32^ BAFF promotes the survival, proliferation, and differentiation of B lymphocytes by binding to B cell activator receptor, transmembrane activated calmodulin ligand and B cell maturation antigen. BAFF overexpression can cause severe autoimmune diseases. Studies have shown that levels of BAFF and its mRNA are significantly elevated in the cytoplasm of patients with ITP. Zhu et al demonstrated that BAFF may play a pathogenic role in ITP by promoting the survival of CD19t B cells. Blockage of BAFF by a decoy receptor (BR3‐Fc) reduced BAFF expression, suggesting that this type of blockage might be a promising therapy for ITP.^33^ These studies indicate that B cells are another therapeutic target for the treatment of ITP in addition to T cells. Li et al studied 36 patients with chronic ITP, and found that the function of regulatory B (Breg) cells and the activation of B lymphocytes by IL‐10 were impaired, and Breg cells were associated with the efficacy of thrombopoietin. Therefore, Breg cells, like regulatory T cells (Treg), have the effect of maintaining peripheral immune tolerance and mitigating immune responses, both of which play a similar role in the development of ITP.

The goal of primary ITP therapy is to increase the patient's platelet count to a safe level and reduce the mortality rate, but excessive treatment should be avoided as much as possible. Emergency treatment of ITP is usually used for active bleeding in important areas or for patients requiring emergency surgery. In addition, thromboproliferative drugs have been added to the emergency treatment of ITP. As of now, first‐line treatment of ITP includes glucocorticoids and intravenous high‐dose gamma globulin.^34^ The corticosteroid non‐responsive refractory ITP may require splenectomy.^35^ Thrombopoin‐promoting drugs are the preferred second‐line treatment for ITP, including recombinant human thrombopoietin (rhTPO), atratropipin, and romitastatin, all supported by prospective multicenter randomized controlled clinical data. These drugs take effect quickly (1 to 2 weeks), but their efficacy is generally not maintained after discontinuation of the drug, and individualized maintenance is required. Although these treatments generally have better therapeutic effects, clinical trials have shown that the above treatments have certain side effects, including thrombosis, bone marrow reticulum hyperplasia, bone marrow collagen hyperplasia, and the production of neutralizing antibodies but not endogenous TPO.^36^ In contrast, the traditional Mongolian medicine QSBLE, a formula made from natural products, can effectively treat ITP and, more importantly, appears to have no obvious side effects after treatment.^10^ An in‐depth understanding of the changes in protein levels and molecular mechanisms after QSBLE treatment in ITP, such as is provided by nano‐LC/MS/MS analysis, will facilitate the widespread use of the drug and the precise treatment of the disease.

With the advances in proteomics technology, and separation, purification, and identification technologies, the use of proteomics to identify clinical markers has attracted a lot of attention. The technology of proteomics research is diverse, and it is helpful in all aspects of biomarker research.^37^, ^38^, ^39^ Proteomics also has great potential in the study of neoplastic diseases. However, research on ITP is still rare. Only a limited number of studies have reported proteomics‐based findings on ITP, although there is a recent study on the proteome of ITP and MS‐based antibody‐mediated identification of autoantigens (MS‐AMIDA) for platelet antigens.^40^

In this study, we identified a total of 982 differentially expressed proteins in ITP compared with controls, using iTRAQ labeling and nano‐LC/MS/MS analysis. Based on a change >1.2‐fold, 48 proteins were upregulated and 213 were downregulated after effective QSBLE treatment compared with pre‐QSBLE treatment (Table 2). The data for the invalid group are also listed in Table 2; they were not directly related to the effect of QSBLE on the treatment of ITP, but they do have potential research value. After identifying significant differentially expressed proteins, we performed bioinformatics analysis to further extract information from the proteomics data. GO analysis provided insights into the molecular functions and biological processes of relevant ITP proteins, and pathway enrichment analysis was also performed by searching against the KEGG database. According to the results of KEGG enrichment analyses, 29 pathways were significantly enriched. The KEGG enrichment analyses revealed that these differentially expressed proteins were highly associated with immune diseases, and immune system development and function, such as the Fc epsilon RI signaling pathway and immune network for IgA production. We further screened out 20 proteins which are expressed in normal people as well as in pre‐ and post‐QSBLE treatments in ITPs. Of these 20 proteins, MIF, PGK1, and IGHM are closely related to immunity.^18^, ^19^, ^20^ As ITP is an autoimmune disorder characterized by autoantibody production against platelets and increased platelet destruction, platelets can both supply and respond to signals at the early stages of immune control.^41^ The results of GO analysis also showed that the differentially expressed proteins are involved in the biological process of adhesion, response to stimulus and immune system processes and molecular function of molecular transducer activity, binding and catalytic activity, cellular component of cell junction, and macromolecular complex (Figure 2). The above‐mentioned results imply that MIF, IGHM, and PGK1 may affect the function of platelets which are highly related to ITP. Overexpression of the miR‐98‐5p can inhibit the PI3K/Akt signaling pathway and impair the therapeutic effect of MSCs in ITP mice.^42^ Furthermore, the PPAR and NF‐κB signaling pathways are involved in regulating ITPs.^43^ The PI3K/Akt, PPAR, and NF‐κB signaling pathways related to ITP are also those that are highly enriched by the 20 differentially expressed proteins described here (Figure 3). Although the pathways and biological processes of these differentially expressed proteins have been suggested to be enriched and involved, the relationship between the proteins and the miRNAs that we previously reported still needs to be further studied.^11^ ITP is a rare disease, our clinical screening standards were very strict, and the number of patients with effective treatment drugs will be further reduced. Thus, the number of patients ultimately available is very limited. The data from this study show differentially expressed proteins between ITP patients and normal people to a certain extent in Inner Mongolia. In addition, before putting these results into clinical use, it is necessary to further verify the stability and pathways of the identified differentially expressed proteins by *in vitro* experiments, analyze the role of these proteins in the pathogenesis of ITP, and further test the safety, reliability, and effectiveness of the results through animal experiments *in vivo*.

5

### PEER REVIEW

The peer review history for this article is available at https://publons.com/publon/10.1002/rcm.8993.

## Supporting information


**Table S1.** Primers for real‐time PCR Note: The sequence refers to the primers for the real‐time PCR.
**Table S2.** The clinical information of each patient.
**Table S3.** Overview of the gender and age of ITP patients treated with QSBLE.
**Table S4.** Protein identification results of ITP compared with controls.
**Table S5.** Significantly differentially expressed proteins identified by iTRAQ analysis of ITPs before and after QSBLE treatment.

**Table S6.** Peptides identification information including the Peptide‐Spectrum Match (PSM), the [M + H]^+^ precursor ion, and the precursor ion charge.
