# Supplementary material for: iTRAQ‐based quantitative proteomics analysis of immune thrombocytopenia patients before and after Qishunbaolier treatment
Source: Rapid Commun Mass Spectrom. 2020 Dec 3;35(3):e8993. doi: 10.1002/rcm.8993 (PMC7757159; doi:10.1002/rcm.8993)
Supplement: Supplementary file 1 — Table S1. Primers for real‐time PCR Note: The sequence refers to the primers for the real‐time PCR. Table S2. The clinical information of each patient. Table S3. Overview of the gender and age of ITP patients treated with QSBLE. Table S4. Protein identification results of ITP compared with controls. Table S5. Significantly differentially expressed proteins identified by iTRAQ analysis of ITPs before and after QSBLE treatment. [file RCM-35-e8993-s001.docx]

**Supporting information**

**Table S1. Primers for real-time PCR** Note: The sequence refers to the primers for the real-time PCR.

**Table S2. The clinical information of each patient.**

**Table S3. Overview of the gender and age of ITP patients treated with QSBLE.**

**Table S4. Protein identification results of ITP compared with controls.**

**Table S5. Significantly differentially expressed proteins identified by iTRAQ analysis of ITPs before and after QSBLE treatment.**

Table S1

| Gene | Forward primer sequence | Reverse primer sequence |
| --- | --- | --- |
| *Mif* | CGCAGAACCGCTCCTACA | GAGTTGTTCCAGCCCACA |
| *PGK1* | GAACAAGGTTAAAGCCGAGCC | GTGGCAGATTGACTCCTACCA |
| *IGHM* | CTTGTCCCCGGAGAAGTATGT | CGGTGGTACTGTAGAAGAGGC |
| *GAPDH* | GGAGCGAGATCCCTCCAAAAT | GGCTGTTGTCATACTTCTCATGG |

Note: The patients were divided into two groups, with each group comprising 9 people, BT, before treatment; AT, refers after treatment. The unit “×10^9^/L” refers to the concentration of platelets.

Table S2

| Group | Patients | Gender | Age | BT (×10^9^/L) | AT (×10^9^/L) |
| --- | --- | --- | --- | --- | --- |
| Effective group | 1 | Female | 50 | 45 | 96 |
| Effective group | 2 | Male | 35 | 15 | 194 |
| Effective group | 3 | Male | 36 | 65 | 180 |
| Effective group | 4 | Female | 45 | 47 | 75 |
| Effective group | 5 | Female | 52 | 50 | 97 |
| Effective group | 6 | Male | 40 | 62 | 99 |
| Effective group | 7 | Male | 47 | 49 | 99 |
| Effective group | 8 | Male | 26 | 25 | 130 |
| Effective group | 9 | Female | 25 | 64 | 111 |
| Invalid group | 10 | Male | 45 | 26 | 26 |
| Invalid group | 11 | Female | 42 | 61 | 67 |
| Invalid group | 12 | Female | 24 | 33 | 36 |
| Invalid group | 13 | Female | 55 | 72 | 77 |
| Invalid group | 14 | Female | 27 | 21 | 11 |
| Invalid group | 15 | Female | 52 | 78 | 33 |
| Invalid group | 16 | Male | 22 | 22 | 25 |
| Invalid group | 17 | Female | 34 | 45 | 61 |
| Invalid group | 18 | Male | 19 | 3 | 17 |

| Table S3 | | |
| --- | --- | --- |
| Group | Male | Female Age |
| Effective treatment | 5 | 4 39.56±9.9 |
| Invalid  treatment | 3 | 6 35.56±13.45 |

Note: There was no significant difference between the two groups in gender and age (p>0.05).

Table S4

| **Database** | **No.** | **Total spectra** | **Spectra (PSM)** | **Peptides** | **Unique peptides** | **Protein groups** |
| --- | --- | --- | --- | --- | --- | --- |
| HomoSapiens | 1 | 217031 | 28037 | 7874 | 4102 | 732 |
| HomoSapiens | 2 | 228318 | 31484 | 9152 | 4665 | 799 |
| HomoSapiens | 3 | 223904 | 33251 | 9202 | 4680 | 833 |
| HomoSapiens | Total | 669253 | 92934 | 11903 | 5717 | 982 |

Note: Total spectra refer to the total number of secondary mass spectra; Spectra (PSM, Peptide Spectrum Match) refers to identifying the number of spectra matched by peptides.

Table S5

| Accession | | Gene Name | | Average B | | Average D | D/B | P-Value |
| --- | --- | --- | --- | --- | --- | --- | --- | --- |
| Q8NEJ1 | |  | 78.23333±8.6 | | 115.5333±1.6 | 1.476779 | 0.002409 |  |
| A2MYE1 |  | | 74.56667±7.9 | | 98.53333±7.8 | 1.321413 | 0.021073 |  |
| Q01469 | | FABP5 | 77.8±4.2 | | 101.45±2.4 | 1.303985 | 0.00326 |  |
| A8K690 | |  | 72.5±6.2 | | 92.86667±3.2 | 1.28092 | 0.044186 |  |
| Q6N030 | | IGHM | 74.2±7.9 | | 92.46667±3.6 | 1.246181 | 0.001979 |  |
| A0A024QYT5 | | SERPINE1 | 79.93333±3.9 | | 99.5±1.9 | 1.244787 | 0.042353 |  |
| Q9UL89 | |  | 82.63333±0.6 | | 102.8±3.3 | 1.24405 | 0.012854 |  |
| P14174 | | MIF | 50.4±5.3 | | 62.15±4.9 | 1.233135 | 0.048287 |  |
| A8K0T9 | |  | 75.06667±2.3 | | 91.63333±2.8 | 1.220693 | 0.038295 |  |
| P37840 | | SNCA | 73.2±5.6 | | 89.03333±0.6 | 1.216302 | 0.004255 |  |
| Q16610 | | ECM1 | 82.16667±2.4 | | 99.36667±3.4 | 1.209331 | 0.012711 |  |
| P00558 | | PGK1 | 64.63333±5.6 | | 77.8±1.1 | 1.203713 | 0.037871 |  |
| P02671 | | FGA | 71.16667±7.3 | | 58.93333±4.7 | 0.828103 | 0.013528 |  |
| A0A140VKA6 | |  | 65.65±3.6 | | 54.05±10.2 | 0.823305 | 0.020774 |  |
| P02679 | | FGG | 55.9±1.2 | | 45.56667±1.7 | 0.815146 | 0.02196 |  |
| B4E0X1 | |  | 115.6333±6.4 | | 90.43333±4.3 | 0.78207 | 0.002886 |  |
| P23528 | | CFL1 | 59.76667±6.0 | | 45.76667±11.1 | 0.765756 | 0.001329 |  |
| P24298 | | GPT | 133.35±3.4 | | 97.3±7.4 | 0.729659 | 0.027884 |  |
| A2VCK8 | | TMSB4X | 48.63333±4.8 | | 34.9±5.4 | 0.717615 | 0.000246 |  |
| B4DPU2 | |  | 142.2±3.6 | | 50.1±2.4 | 0.352321 | 0.002552 |  |
| Q7Z7Q0 | | APOB | 66.63333±10.2 | | 125.13333±6.2 | 1.877939 | 0.045326 |  |
| P25815 | | S100P | 72.05000±5.8 | | 128.20000±37.6 | 1.779320 | 0.047257 |  |
| Q6N092 | DKFZp686K18196 | | 79.10000±1.2 | | 139.83333±1.3 | 1.767804 | 0.000006 |  |
| A0A024QZV1 | | PLK2 | 78.63333±6.1 | | 138.40000±2.5 | 1.760068 | 0.026819 |  |
| H7C0L5 | | ITIH4 | 80.60000±10.9 | | 124.26667±6.6 | 1.541770 | 0.003248 |  |
| P32942 | | ICAM3 | 79.40000±9.6 | | 120.20000±0.3 | 1.513854 | 0.017281 |  |
| A2KUC4 | | IGLV | 71.53333±3.0 | | 105.33333±7.6 | 1.472507 | 0.025007 |  |
| A0A024R3E3 | | APOA1 | 89.73333±9.1 | | 126.83333±4.1 | 1.413447 | 0.033902 |  |
| Q9UL82 | |  | 78.20000±22.6 | | 109.40000±9.8 | 1.398977 | 0.000733 |  |
| A2N7P4 | | IGHM | 73.20000±5.1 | | 102.16667±2.7 | 1.395719 | 0.014255 |  |
| Q9UL85 | |  | 82.33333±7.9 | | 113.06667±17.1 | 1.373279 | 0.002896 |  |
| A0A0F7TAV4 | | IGHV5-51 | 81.73333±3.4 | | 112.03333±5.8 | 1.370718 | 0.014616 |  |
| A0A1C9J6R5 | |  | 87.13333±20.5 | | 116.80000±0.6 | 1.340474 | 0.026454 |  |
| A6H8L5 | | LIPC | 70.95000±13.2 | | 94.85000±12.9 | 1.336857 | 0.045084 |  |
| B2R892 | |  | 89.30000±10.2 | | 118.35000±2.9 | 1.325308 | 0.030906 |  |
| B4DTH2 | |  | 86.63333±5.9 | | 114.80000±2.7 | 1.325125 | 0.000658 |  |
| Q59G48 | |  | 85.50000±16.7 | | 112.55000±16.1 | 1.316374 | 0.022598 |  |
| S6AWF4 | |  | 81.63333±14.2 | | 107.30000±9.5 | 1.314414 | 0.000083 |  |
| A0A075B7B8 | | IGHV3OR16-12 | 82.56667±9.0 | | 107.73333±1.5 | 1.304804 | 0.002761 |  |
| A0A193CHQ9 | |  | 81.46667±10.3 | | 106.26667±10.4 | 1.304419 | 0.041977 |  |
| A0A024RD26 | | GPR116 | 85.10000±9.9 | | 110.96667±6.6 | 1.303956 | 0.001844 |  |
| A0A0B4J1X5 | | IGHV3-74 | 84.86667± | | 6.2109.96667±0.4 | 1.295758 | 0.008699 |  |
| A0A0X9UWL5 | |  | 82.23333±2.0 | | 105.43333±4.8 | 1.282124 | 0.034079 |  |
| P24043 | | LAMA2 | 82.75000±5.5 | | 105.85000±2.1 | 1.279154 | 0.022432 |  |
| H3BUX1 | | MSLN | 85.50000±4.5 | | 108.90000±13.2 | 1.273684 | 0.012119 |  |
| A0A024QZP2 | | SFTPA1 | 87.45000±1.1 | | 110.40000±2.3 | 1.262436 | 0.029169 |  |
| Q9UL78 | |  | 84.13333±5.0 | | 105.66667±1.2 | 1.255943 | 0.012850 |  |
| Q5NV90 | | V2-17 | 80.50000±1.7 | | 100.13333±29.3 | 1.243892 | 0.002867 |  |
| Q6PIQ7 | | IGL@ | 80.53333±5.1 | | 99.26667±0.9 | 1.232616 | 0.007767 |  |
| A0A0X9TD47 | |  | 86.60000±2.2 | | 106.13333±0.6 | 1.225558 | 0.043396 |  |
| A0A0X9TD88 | |  | 88.56667±4.9 | | 108.06667±1.3 | 1.220173 | 0.004394 |  |
| Q6MZQ6 | | DKFZp686G11190 | | 88.86667±1.5 | 108.06667±4.8 | 1.216054 | 0.018386 |  |
| Q9UMV1 | | C4B | 92.10000±8.2 | | 111.83333±5.1 | 1.214260 | 0.016226 |  |
| O15394 | | NCAM2 | 87.43333±6.0 | | 106.10000±3.6 | 1.213496 | 0.021463 |  |
| A0A087WW89 | | IGHV3-72 | 87.53333±3.6 | | 105.20000±1.0 | 1.201828 | 0.002501 |  |
| Q9NQ79 | | CRTAC1 | 92.80000±5.6 | | 111.40000±2.0 | 1.200431 | 0.028337 |  |
| P12318 | | FCGR2A | 105.26667±10.4 | | 85.26667±4.1 | 0.810006 | 0.034462 |  |
| D6RF20 | | GC | 105.16667±5.1 | | 81.93333±14.9 | 0.779081 | 0.043324 |  |
| A0A0D9SG04 | | COBLL1 | 110.20000±3.3 | | 80.10000±2.9 | 0.726860 | 0.003797 |  |
| B2R773 | |  | 121.80000±12.1 | | 86.60000±6.4 | 0.711002 | 0.041094 |  |
| P25789 | | PSMA4 | 130.85000±7.2 | | 67.05000±4.2 | 0.512419 | 0.041996 |  |

Note: Accession refers to protein accession number; Average refers to average relative expression of corresponding group proteins; D/B refers the averageB/ averageD, P-Value refers to the statistical test of relative expression of protein between two groups of samples. Each sample contained the blood of 3-5 individuals. The test was repeated three times for each sample. Statistical data are presented as mean ± SEM. *P < 0.05; **P < 0.01.
