# Supplementary material for: iTRAQ‐based quantitative proteomics analysis of immune thrombocytopenia patients before and after Qishunbaolier treatment
Source: Rapid Commun Mass Spectrom. 2020 Dec 3;35(3):e8993. doi: 10.1002/rcm.8993 (PMC7757159; doi:10.1002/rcm.8993)
Supplement: Supplementary file 2 — Table S6. Peptides identification information including the Peptide‐Spectrum Match (PSM), the [M + H]+ precursor ion, and the precursor ion charge. [file RCM-35-e8993-s002.docx]

**Table S6. Peptides identification information** including the Peptide-Spectrum Match (PSM), the [M+H]^+^ precursor ion, and the precursor ion charge**.**

Note: PSM refers to Peptide-Spectrum Match.
